# Reply to Andreas Boehle, Frank Kahmann, Thomas Oliver Henkel, Joerg Zimmermann and Stefan Machten’s to the Letter to the editor Re: results of a randomized trial of treatment modalities in patients with low or early-intermediate risk prostate cancer (PREFERE trial)

**DOI:** 10.1007/s00432-021-03549-y

**Published:** 2021-02-24

**Authors:** Thomas Wiegel, Peter Albers, Detlef Bartkowiak, Roswitha Bussar-Maatz, Martin Härter, Glen Kristiansen, Peter Martus, Stefan Wellek, Heinz Schmidberger, Klaus Grozinger, Peter Renner, Fried Schneider, Martin Burmester, Michael Stöckle

**Affiliations:** 1 Department of Radiotherapy and Radiation Oncology, University Hospital Ulm, Albert-Einstein-Allee 23, 89081 Ulm, Germany; 2 Department of Urology, University Hospital Düsseldorf, Düsseldorf, Germany; 3 PREFERE Project Management, German Cancer Society, Berlin, Germany; 4 Department of Medical Psychology, University Medical Center Hamburg-Eppendorf, Hamburg, Germany; 5 Institute of Pathology, University Hospital Bonn, Bonn, Germany; 6 Department of Clinical Epidemiology and Applied Biostatistics, University Hospital Tübingen, Tübingen, Germany; 7 Department of Medical Biostatistics, Epidemiology and Informatics, University of Mainz, Mainz, Germany; 8 Department of Radiotherapy and Radiation Oncology, University Hospital Mainz, Mainz, Germany; 9 Department of Urology, Klinikum Leverkusen, Leverkusen, Germany; 10 Center for Urology, Lübeck, Germany; 11 Department of Urology, Klinikum Lippe Detmold, Detmold, Germany; 12 Department of Urology, Vinzenzkrankenhaus, Hannover, Germany; 13 Department of Urology, University Hospital Homburg/Saar, Homburg, Germany

We thank the colleagues A. Boehle, F. Kahmann, T.O. Henkel, J. Zimmermann and S. Machtens for their remarks (Boehle et al. 2020).

First, we acknowledge the meticulous review of our paper and we revised the graphical errors in figure 3 referring to acute and 12-months toxicities of the treatment options. In addition, we have re-analyzed those toxicity data and provide a revised figure (Wiegel et al. 2020).

Second, the main message of our report is the unexpectedly high rate of termination of AS and the observation of a numerical higher toxicity rate of PSI versus EBRT. Details of patients with AS within PREFERE have been reported elsewhere (Albers et al. 2020). The higher number of toxicities and the results of the quality assurance of the PSI-patients was indeed surprising and stimulated us to report on these observations in more detail (2020). Whether this observation will be indeed true on the long term will be a matter of a further analysis with longer follow-up.

Third, the PREFERE trial was the first randomized trial in prostate cancer which included PSI as a treatment option. We are grateful that most of the authors of the “Letter to the Editor” considerably contributed to this trial by enrolling patients or performing PSI (Stockinger et al. 2020). This gives us the opportunity to report on toxicities and efficacy of PSI within a prospectively controlled setting including details on quality assurance of every treatment option performed. Therefore, long-term data on oncological efficacy including PSI can be reported at the time of sufficient follow-up. As agreed with all contributors of PREFERE, oncological outcomes of all treatment options in patients with localized prostate cancer may be reported only with at least 10 years follow-up and intentionally were not matter of debate of this report (Neal et al. 2020).

Finally, our report on preliminary data of a prematurely closed trial definitively did not intend to subjectively favor one over the other treatment option as suggested by Boehle et al. We unfortunately had to state that the optimal treatment of low and early intermediate PCa still remains unclear.
